# Atypical Rapunzel Syndrome in an Adult Female: Trichobezoar Beyond the Gastric Outlet Leading to Gastrointestinal Perforation and Septic Shock

**DOI:** 10.7759/cureus.88436

**Published:** 2025-07-21

**Authors:** Amber R Jacobson, Lynnsey M Rebner, Hamdan Mallick, Krishnaraj Mahendraraj, Theodoros Katsichtis

**Affiliations:** 1 General Surgery, Bayhealth Medical Center, Dover, USA; 2 Medicine, Bayhealth Medical Center, Dover, USA; 3 Hepatobiliary Surgery, Bayhealth Hospital, Dover, USA; 4 Bariatric Surgery, Bayhealth Hospital, Dover, USA

**Keywords:** bezoar, gastric trichobezoar, rapunzel syndrome, trichophagia, trichotillomania

## Abstract

Trichobezoars are rare accumulations of ingested hair typically seen in young females with psychiatric disorders such as trichotillomania and trichophagia. Few case reports describe separate trichobezoar masses within the GI tract. We report the case of a 57-year-old female with trichophagia history who presented with abdominal distensions and rebound tenderness consistent with peritonitis. Imaging raised suspicion for bezoars, and emergent laparotomy revealed two separate trichobezoars, one in the gastric antrum and one in the jejunum, as well as two jejunal perforations. This case highlights that trichobezoars can present in an atypical demographic and extend beyond the gastric outlet, potentially resulting in life-threatening complications requiring prompt surgical management.

## Introduction

Trichobezoars, also known as ingested hair, are rare but fascinating clinical entities predominantly found in the gastrointestinal tract, typically the stomach. Their occurrence in humans is linked to psychiatric conditions, particularly trichotillomania (compulsive hair pulling) and trichophagia (compulsive hair eating), and is more commonly seen in countries where women have traditionally longer hair [1,2]. Most reported cases involve adolescents or young adults. According to Pipal et al., the mean age of trichobezoar cases is 11 years, and they have a sevenfold higher prevalence in children than adults, primarily affecting females [3].

The formation of trichobezoars can lead to significant clinical symptoms, ranging from abdominal pain and nausea to more severe complications such as gastric outlet obstruction, perforation, and peritonitis [4]. Other notable complications include intussusception, pancreatitis and cholangitis [5]. Even rarer are gastric trichobezoars with a tail that traverses past the pylorus, known as “Rapunzel syndrome,” characterized by Vaughan et al. in 1968 [6]. They come in various sizes and one of the longest trichobezoars to be documented was 59 inches long which was managed operatively [7].

Management of trichobezoars generally requires a multidisciplinary approach, combining surgical intervention for the removal of the mass with psychiatric treatment to address the underlying behavioral issues. In this report, we present a unique case of a 57-year-old female found to have a trichobezoar in two separate locations, as well as two separate small bowel perforations during an exploratory laparotomy for peritonitis. By documenting this case, we aim to highlight the unique situation of a middle-aged female (with history of trichophagia as a teenager) who had two unconnected trichobezoars causing obstruction, perforation, and peritonitis necessitating two separate enterotomies for removal.

## Case presentation

**Figure 1:**
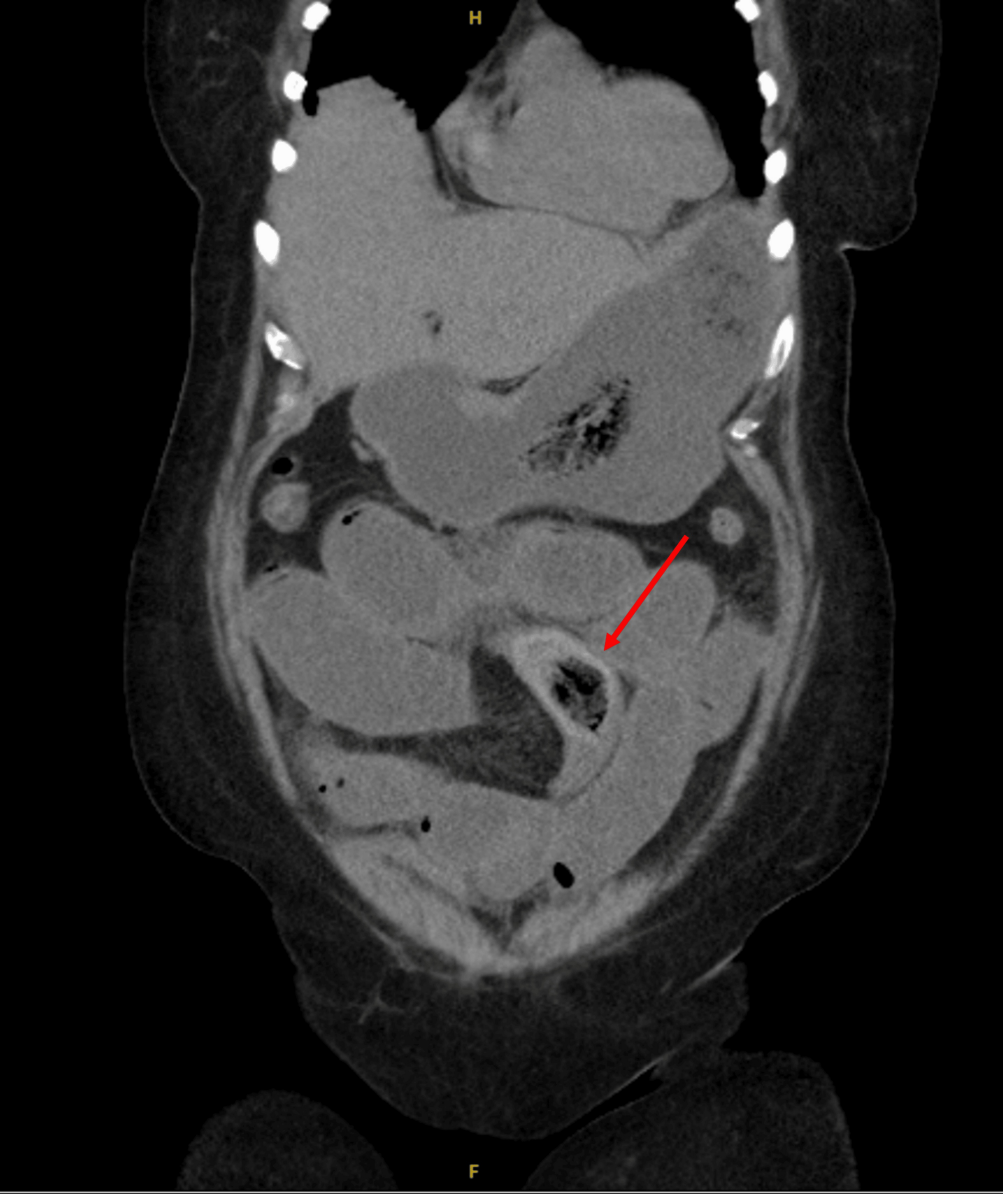
Non-contrast CT scan, coronal view, displaying a small bezoar seen in the mid abdomen (red arrow).

**Figure 2:**
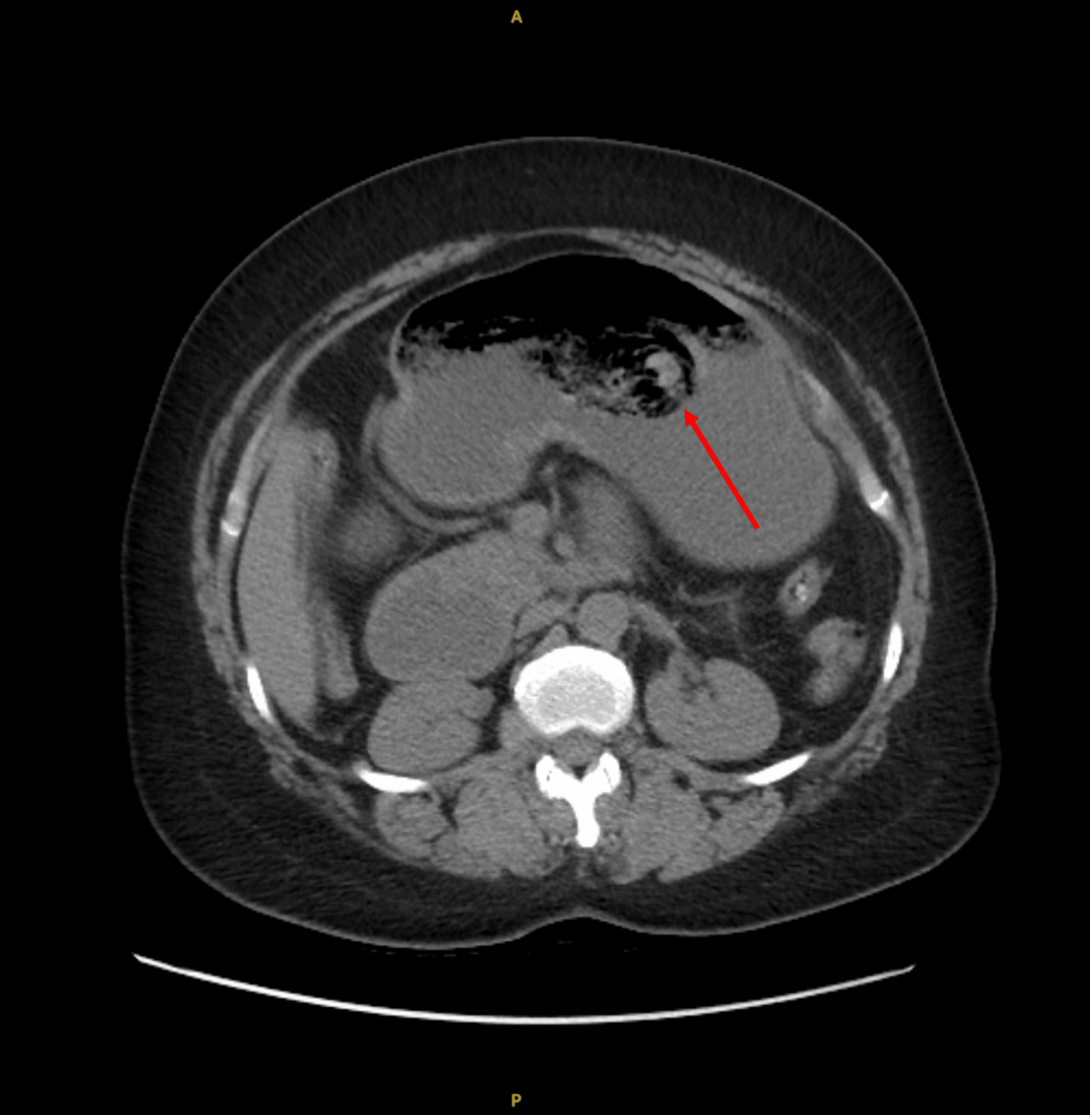
Non-contrast CT scan, axial view, showcasing a large bezoar mass in the gastric antrum (red arrow).

The patient is a 57-year-old female with a past medical history of hypertension, hyperlipidemia, partial hysterectomy, and remote history of trichophagia as a teenager who presented to the emergency department with significant abdominal pain, malodorous breath/belching, and constipation. She was hypotensive at the time but otherwise afebrile and non-tachycardic. On exam, the patient appeared uncomfortable with dry mucous membranes as well as abdominal distension and rebound tenderness consistent with peritonitis. Significant labs at the time included hyponatremia, an elevated anion gap, acute kidney injury, and iron deficiency anemia. No leukocytosis or elevated lactate was seen (Table 1). On a non-contrast computed tomography (CT) scan, there appeared to be a poorly defined, heterogeneous, low to intermediate attenuated mass within the gastric body and small bowel which raised concern for a bezoar (Figure 1, 2). 

**Table 1:** Patient's pertinent laboratory values at initial presentation. (L): Data is abnormally low (H): Data is abnormally high

	Reference Range & Units	Results
Sodium	136 - 145 mmol/L	120 (L)
Anion Gap	5.0 - 15.0 mmol/L	20.0 (H)
BUN	7 - 18 mg/dL	176 (H)
Creatinine Serum	0.6 - 1.0 mg/dL	9.0 (H)
eGFR	>60 mL/min/1.73m*2	7 (L)
Iron	50 - 170 ug/dL	21 (L)
Lactate, Ven	0.4 - 2.0 mmol/L	1.4
WBC	4.5 - 11.0 K/µL	9.9
Hemoglobin	12.0 - 16.0 g/dL	10.4 (L)
Hematocrit	37.0 - 47.0 %	29.4 (L)
Platelets	150 - 400 K/uL	347

Given her peritonitis on exam as well as concerning findings on CT, she was taken to the operating room for an emergent exploratory laparotomy. Intraoperatively, purulent peritonitis was discovered with gross enteric spillage. After lavage, the bowel was run from the ligament of Treitz to the ileocecal valve and the stomach was examined. There were two palpated intraluminal masses, one just proximal to the pylorus in the stomach and another in the mid-jejunum. Two jejunal perforations adjacent to the mesentery were noted proximal to the mid-jejunal mass, measuring 3 cm each. There was no evidence of necrotic bowel.

**Figure 3:**
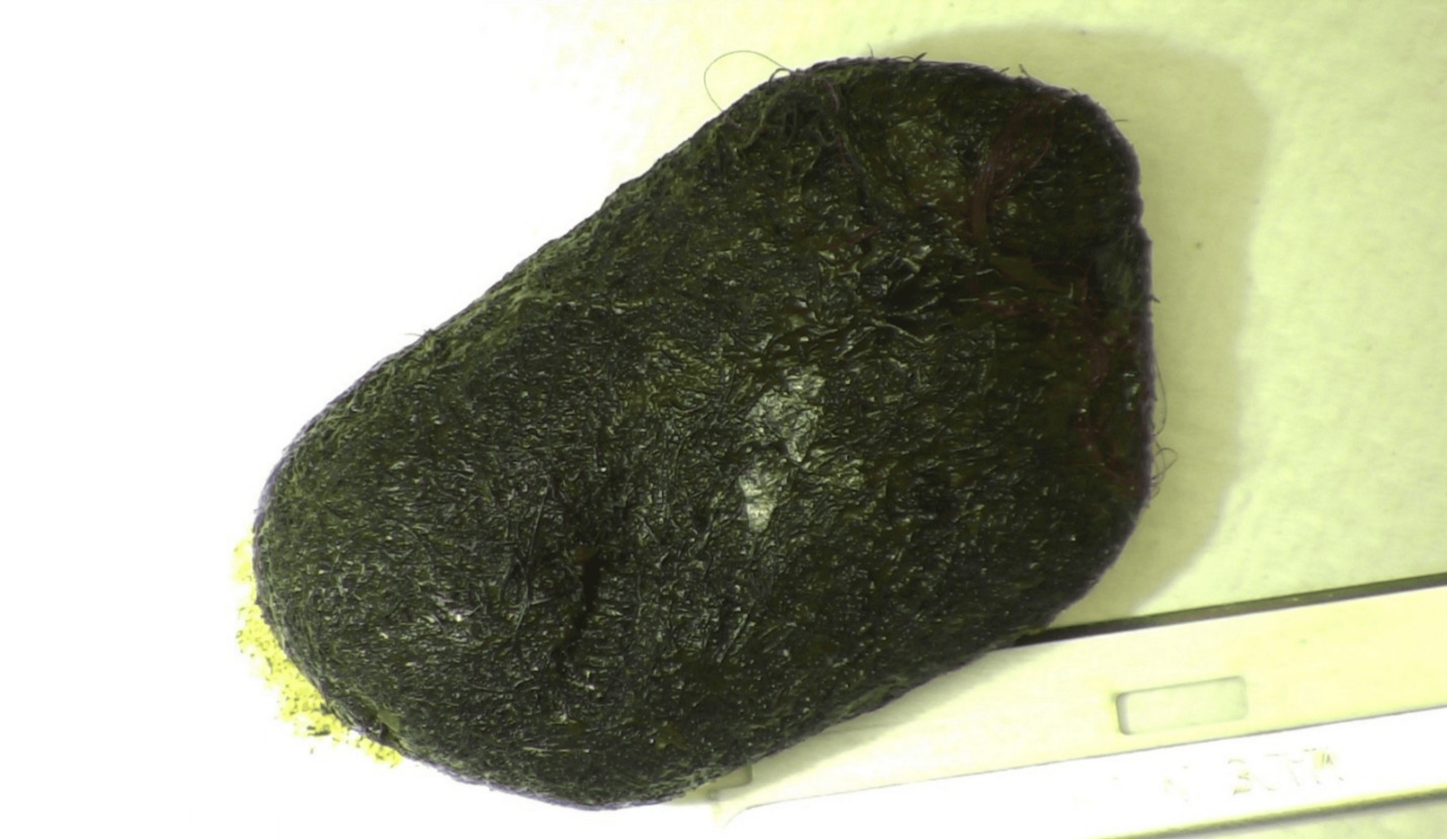
Gross pathology photograph showcases one of the bezoars that was extracted from the bowel.

**Figure 4:**
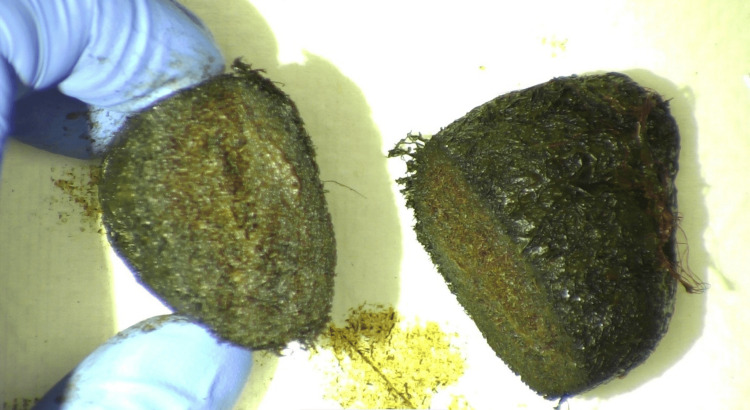
Gross pathology photograph displaying the same bezoar mass opened to reveal hair fibers at the center of the mass.

**Figure 5:**
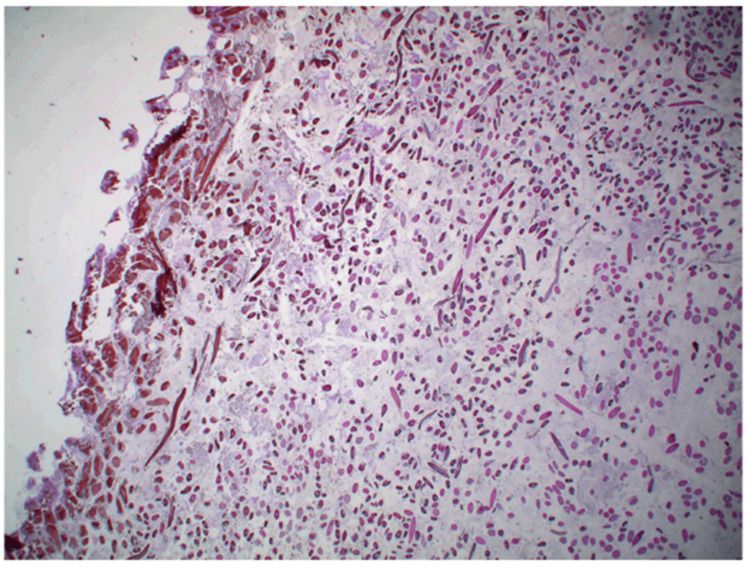
Microscopic examination with hematoxylin and eosin stain revealed fibrous material without adherent soft tissue. 20x magnification.

**Figure 6:**
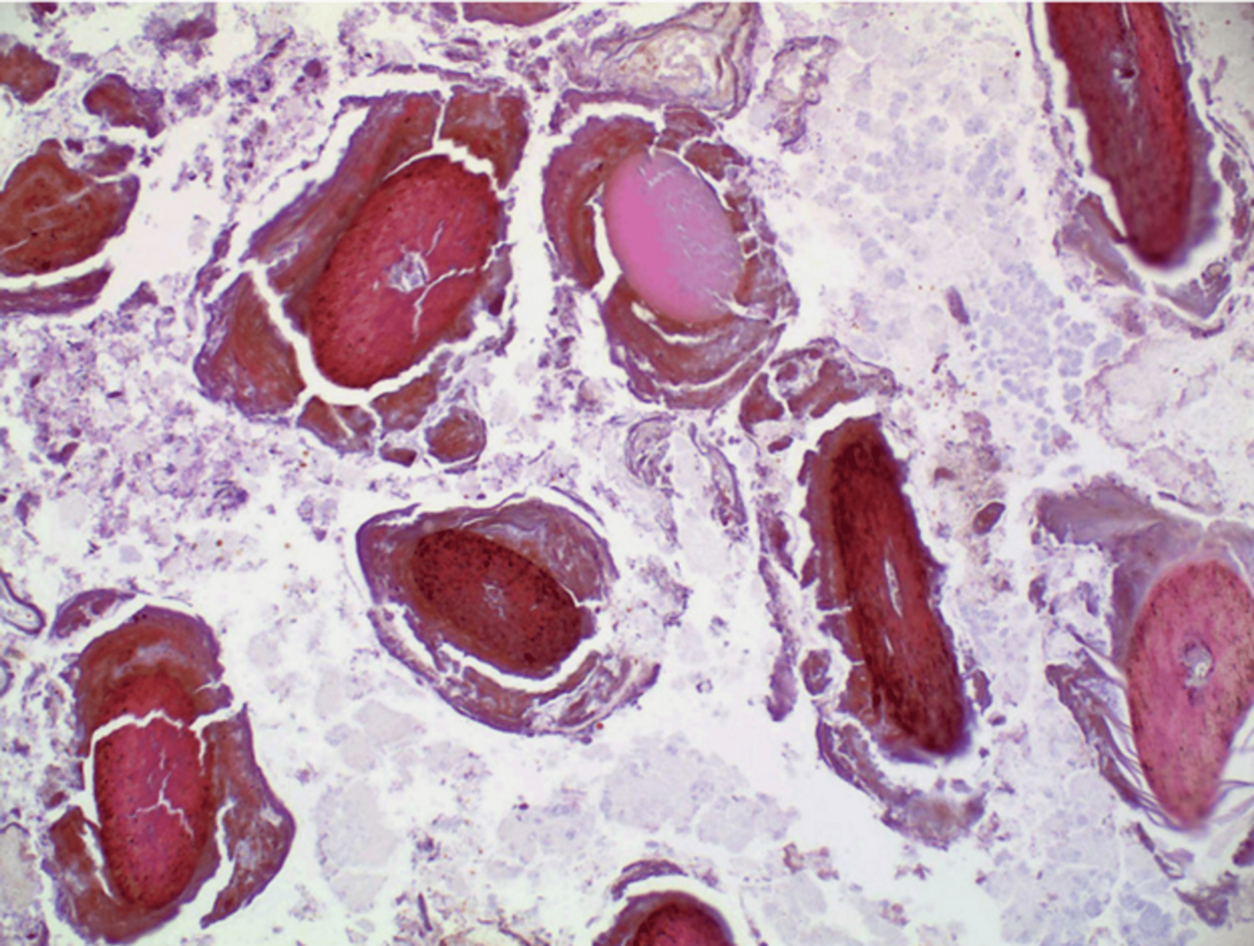
Microscopic examination with hematoxylin and eosin stain revealing tightly packed, coarse, keratinized hair fibers that are interwoven in a haphazard fashion with well-formed keratin pearls consistent with hair. 40x magnification.

The intraluminal masses were explored via gastric and jejunal enterotomies. A small longitudinal enterotomy was made proximal to the jejunal mass and it was milked retrograde and removed. Once removed, the mass appeared to be a trichobezoar. The gastric mass was milked retrograde from the pre-pyloric region and appeared similar to the trichobezoar from the jejunum. The stomach mass measured 10 x 4 x 4 cm, and the jejunal mass measured 5.8 x 3.5 x 2.2 cm, both consistent with trichobezoar on pathology exam (Figures 3-6).

Both perforations and enterotomies were closed with 3-0 V-Loc suture transverse to the long axis of the bowel, in Heineke-Mikulicz fashion with additional Lembert sutures. Her abdomen was lavaged again with copious irrigation. Given her hemodynamic instability and increasing pressor requirements, the decision was made for temporary abdominal closure with an AbThera wound vacuum.

A second-look laparotomy was performed on postoperative day two. Both small bowel perforation repairs, as well as the gastrostomy and enterotomy incisions that were made to remove the bezoars, were all intact without evidence of leak. Her abdominal wall was bridged and closed with biologic mesh.

In the immediate postoperative course, she remained intubated and sedated with vasopressor requirements and continuous renal replacement therapy for severe acute kidney injury and ongoing septic shock. Her blood culture was positive for Candida glabrata, likely secondary to contamination from the small bowel perforations, and she was treated with broad-spectrum antibiotics and antifungals. With treatment, she subsequently stabilized, extubated, transitioned to hemodialysis, and tolerated a diet. She was evaluated by psychiatry and admitted to trichophagia during her teenage years due to anxiety, but denied any recent trichophagia. Her zinc, copper, and selenium levels were all normal. The patient continued to improve and was eventually discharged to acute rehab. She followed up with general surgery four months post-op and denied any further trichophagia or gastrointestinal problems. 

## Discussion

Trichobezoars are rare cases of accumulated ingested hair that withstand digestion due to their smooth, enzyme‐resistant properties, allowing them to become enmeshed with food and mucus in the stomach [1]. Gastric rugae further trap hair fibers and gastric churning facilitates these fibers to form a bezoar [2,8]. These bezoars can then develop into considerable sizes and may extend beyond the stomach, known as Rapunzel syndrome, which was first characterized by Vaughan et al. in 1968 [6]. They may also migrate beyond the stomach and are frequently found within 50-70 cm of the ileocecal valve, where the lumen narrows, motility decreases, and increased water absorption reduces the mobility of intestinal contents [9]. Additionally, factors that cause delayed gastric emptying, such as prior gastric surgery, vagotomy, pyloroplasty, peptic ulcer disease, chronic gastritis, Crohn's disease, GI malignancy, hypothyroidism, or even dehydration can exacerbate trichobezoar formation [1,8]. These underlying factors may be present in older patients with trichobezoar. 

Classically, these masses are encountered in young females with psychiatric conditions such as trichotillomania and trichophagia, with multiple cases reporting trichobezoars in the pediatric population [10,11]. In 90% of cases, patients are predominantly female, and typically before the age of 30 in 80% of cases [11]. Alopecia is also commonly seen concurrently as in one case report [5]. In contrast, our patient, a 57‐year‐old female, represented an atypical presentation regarding both age as well as the presence of two separate trichobezoars causing perforation. Our patient did not have any known alopecia but did admit (postoperatively) to a remote history of trichophagia when she was a teenager. Despite denying recent trichophagia, the formation of multiple trichobezoars suggests ongoing or intermittent behavior that may have gone unrecognized.

Diagnosis is critical to treat patients with trichobezoars. Along with pertinent patient history, multiple imaging studies are available to aid in diagnosis. Abdominal CT scans can reveal a variable-sized mass with a heterogeneous appearance that occupies a significant portion of the gastric lumen. This mass consists of concentric circles of different densities, resembling an “onion bulb” structure. Per Belhadj et al., two pathognomonic and consistent signs are typically observed: the presence of small air bubbles scattered within the mass and the absence of any adhesion to the gastric wall [11]. Our patient's CT scan presented similarly. Patients can also be diagnosed with barium swallow or endoscopy if they are hemodynamically stable and CT imaging is unclear [4]. 

Some trichobezoars have reached considerable sizes, up to 24 and 27 cm in length in two reports resulting in Rapunzel syndrome [12,13]. Although the trichobezoars in our patient (10 × 4 × 4 cm in the stomach and 5.8 × 3.5 × 2.2 cm in the jejunum) are relatively modest in comparison, each bezoar was found contained without a tail. The presence of two distinct masses is not commonly seen in other case reports. This may be due to the patient consuming hair at different time intervals, and underscores the importance of evaluating for additional bezoars intraoperatively. 

The occurrence of perforation in association with trichobezoars is well documented. Bezoars can impact the bowel wall causing pressure necrosis. Early stages can result in micro-GI bleeding leading to microcytic anemia, and late stages lead to eventual destruction of the bowel wall layers [1]. Our patient was found to have iron deficiency anemia preoperatively, which may reflect a chronic micro-GI bleed due to the trichobezoar, although this cannot be confirmed. Sert et al. described jejunal perforation due to ulceration adjacent to a trichobezoar, and Ahmad et al. reported perforations of the pre-pyloric stomach in a 30‐year‐old woman [5,14]. In both cases, hair and mucous were noted to be extruding from these bowel perforations. In Paparoupa et al., they report a case of a patient with concurrent peptic ulcers adjacent to a bezoar seen on endoscopy [12]. Other perforated bezoar cases are more commonly seen in phytobezoars (bezoars with accumulated vegetable matter) [9]. Our patient's perforations were interestingly proximal and not adjacent to her trichobezoar. This highlights the need to carefully evaluate the small bowel from the ligament of Treitz to the ileocecal valve as bowel perforations are not necessarily in immediate proximity to a bezoar. 

Regarding management, endoscopic removal has been reported to be successful in select cases of small trichobezoars, with success rates approaching 86-89% in highly selected patients [13]. In cases with large or multiple bezoars, or when perforation is present, surgical intervention via laparotomy remains the treatment of choice [4,12]. There are case reports of bezoars removed via laparoscopy; however, these were done in hemodynamically stable patients without generalized peritonitis [2]. Coca-Cola and cellulase are also known to help dissolve phytobezoars, but no substance is known to successfully help dissolve trichobezoars [1]. In our patient, the intraoperative discovery of two perforations, intraperitoneal contamination, all within the setting of septic shock, necessitated the removal of both trichobezoars via open abdominal surgery with enterotomies. 

A multi-disciplinary approach is needed to effectively treat those with trichophagia. Psychiatry was consulted postoperatively once pathology confirmed the bezoars were comprised of hair. Psychiatric management generally consists of psychotherapy or pharmacotherapy and treatment of concurrent psychiatric conditions, such as anxiety, depression, and other mood disorders, however our patient denied any such conditions [14]. Along with psychiatry, a gastroenterologist or nutritionist can be a great addition to work up micronutrient deficiencies associated with pica disorders (the craving and purposeful consumption of non-food substances) such as trichophagia. In a meta-analysis, pica was found to be associated with lower hemoglobin and lower plasma zinc [15]. Although the patient's zinc, copper, and selenium levels were normal, she did present with iron deficiency anemia, which may have exacerbated trichophagia symptoms. 

## Conclusions

In summary, although trichobezoars are predominantly reported in young patients with classic psychiatric disorders, this case illustrates that older patients with trichophagia history may continue to engage in trichophagia until late adulthood, with potentially severe complications such as gastrointestinal perforation, sepsis, and multi-organ dysfunction. Early recognition, prompt imaging evaluation, and decisive surgical management are essential to improve outcomes. Given the atypical age of presentation and the multifocal nature of the bezoars in our patient, this case expands the clinical spectrum of trichobezoar-related pathology. Furthermore, it reinforces the need for postoperative psychiatric and nutritional evaluation with long-term follow-up to minimize recurrence, regardless of patient age. 
